# Incorporating Postbiotics into Intervention for Managing Obesity

**DOI:** 10.3390/ijms26115362

**Published:** 2025-06-03

**Authors:** Emília Hijová, Izabela Bertková, Jana Štofilová

**Affiliations:** Center of Clinical and Preclinical Research-MediPark, Faculty of Medicine, Pavol Jozef Šafárik University, SNP 1, 040 11 Košice, Slovakia; izabela.bertkova@upjs.sk (I.B.); jana.stofilova@upjs.sk (J.Š.)

**Keywords:** obesity, microbiota, postbiotics

## Abstract

Obesity is reaching global epidemic proportions worldwide, posing a significant burden on individual health and society. Altered gut microbiota is considered a key factor in the pathogenesis of many diseases, producing metabolites that contribute to the health-beneficial properties of postbiotics. Postbiotics, bioactive microbial components derived from probiotics, are emerging as a valuable strategy in modern medicine and a promising alternative for managing obesity without the need for live bacteria. This work provides a comprehensive overview of the potential health benefits of postbiotics, particularly in relation to obesity, which represents an important health challenge. Despite the encouraging insights into the health benefits of postbiotics, we highlight the need for further research to clarify the mechanisms and the specific roles of different postbiotic components. Integrating postbiotics into health interventions has the potential to enhance preventive care and significantly improve health outcomes in at-risk populations.

## 1. Introduction

Obesity is a chronic disease with a multifactorial etiology including genetic, environmental, lifestyle, metabolic, and behavioral components. Due to the high prevalence of overweight (pre-obesity) and obesity and the consequent health risks, it has become a major global public health problem accompanied by a range of carbohydrate and lipid metabolism disorders, cardiovascular diseases, certain cancers, and other health conditions [1,2]. The exact mechanisms and interactions underlying metabolic disorders like obesity are complex and not fully understood, leading to ongoing research and debate. While caloric restriction and increased physical activity remain the cornerstones of managing most metabolic disorders, pharmacological treatments are also available and may be necessary, depending on the specific condition [3]. The gut microbiota has been considered essential for health in the last decade [4].

The gut microbiota is a complex community of microorganisms that contributes to metabolic health. Dysbiosis, an imbalance in the gut microbiota, can contribute to metabolic disorders like obesity, impair the effectiveness of synbiotics (probiotics and prebiotics), and deplete beneficial probiotic populations like *Lactobacillus* and *Bifidobacterium* [5]. The gut microbiota is linked to obesity and plays a key role in regulating the fermentation of dietary polysaccharides and fat consumption [6]. Advanced laboratory techniques have enabled the identification and characterization of new biologically active molecules, the so-called “postbiotics” [7,8,9].

The term “postbiotics” refers to a group of bioactive substances derived from non-viable or inactivated microbial cells, their components, or metabolic byproducts of live bacteria (probiotics) that have a beneficial impact on the host [10]. Metabolites produced by the gut microbiota may help alleviate metabolic disorders, including obesity [11], by acting as regulators of energy balance in the host system [12]. Dietary components such as prebiotics are converted into postbiotic metabolites by probiotics or gut microbiota and have been documented as important factors in the treatment of obesity, potentially exerting a positive physiological impact [4,13,14].

Postbiotics, unlike traditional functional foods like probiotics and prebiotics, offer several advantages, including a simpler and more targeted composition, higher physiological activity, longer shelf life, and easier absorption. The administration of live microorganisms carries certain risks and requires careful consideration, especially in individuals with disrupted gastrointestinal microbiota, which may increase susceptibility to disease. To overcome these limitations, the concept of postbiotics has emerged as a promising alternative. Postbiotics are currently being investigated for their potential applications in functional food, pharmaceuticals, and nutraceuticals [15]. Furthermore, postbiotics offer advantages such as greater stability and standardization compared to live probiotics, whose viability and efficacy can vary depending on storage conditions and host. This review highlights the role of postbiotics and presents current knowledge that may support future research into novel postbiotics strategies for nutrition and obesity prevention.

## 2. Gut Microbiota Profile and Obesity-Associated Dysbiosis

Obesity is a chronic, progressive, and relapsing metabolic disease characterized by excessive fat accumulation and impaired mobilization from adipose tissue under normal physiological conditions, with a simultaneous weight gain resulting from an energy imbalance between energy intake and energy expenditure [16]. It is characterized by an increase in body fat exceeding 25% of total body weight in men and 30% in women. According to the World Health Organization (WHO), obesity in adults (aged 18 and older) is defined as a body mass index (BMI) of ≥30 kg/m^2^, while overweight (also referred to as pre-obesity) is defined as a BMI ranging from 25 to 29.9 kg/m^2^. Global estimates indicate that more than 4 billion people could be affected by overweight and obesity by 2035, compared to over 2.6 billion in 2020. This reflects an increase from 38% of the world’s population in 2020 to more than 50% by 2035. In the European Region, the prevalence of obesity among children and adolescents is projected to rise between 2020 to 2035, with 14% of girls and 21% of boys expected to be obese by 2035. In adults, obesity is projected to affect 35% of women and 39% of men by 2035. In Slovakia, the prevalence of obesity in adults is estimated at 31%, with 35% of men and 28% of women expected to be affected by 2035 [3].

Obesity is influenced by physiological, environmental, and genetic factors. Inadequate lifestyle (high-fat diet, sedentary lifestyle, smoking, low physical activity) along with neural and hormonal mechanisms, as well as genetic and epigenetic factors have a significant impact on obesity [17,18]. The gut microbiota also plays a significant role in the prevalence of obesity [19].

Advanced techniques known as “omics” (i.e., metagenomics, metatranscriptomics, metaproteomics, and metabolomics) and bioinformatics tools have significantly contributed to identifying bacterial communities. Bacteroidetes and Firmicutes, along with Actinobacteria, Proteobacteria, and Verrucomicrobia are the most dominant bacteria in the human gut microbiota. These bacteria help maintain body homeostasis by participating in digestion, energy regulation, short-chain fatty acid (SCFA) production, vitamin synthesis, protection against pathogens, and immune system modulation [20]. Changes in the composition of the gut microbiota may play an important role in the pathophysiology of obesity [21]. Obesity contributes to the development of type 2 diabetes, metabolic syndrome, and other related comorbidities, which are accompanied by changes in the gut microbiota. Increased appetite and metabolic dysregulation can disrupt the delicate balance between the gut microbiota and host, leading to persistent dysbiosis. This imbalance may contribute to excessive fat storage and a range of health complications, including obesity and metabolic disorders.

Obese individuals have been shown to exhibit lower diversity and richness in the bacterial composition of the gut microbiota, along with an increased Firmicutes/Bacteroidetes ratio in the fecal microbiota diversity and richness of the bacterial composition of the gut microbiota [22,23,24,25]. The phylum Firmicutes and genera such as *Clostridium*, *Lactobacillus*, or *Ruminococcus* are typically elevated, while *Faecalibacterium prausnitzii* (one of the most abundant bacteria belonging to the Firmicutes phylum in the intestine of healthy individuals) is decreased in people with obesity [26]. Investigations of the gut microbiota in obese individuals have pointed to the presence of obesogenic bacteria, including Firmicutes, Bacteroidetes, *Lactococcus*, *Rhizobium,* and *Clostridium*, as well as bacteria considered beneficial in the context of obesity such as *Lactobacillus* [27]. Despite growing research efforts, no specific bacterial species has been linked to obesity. It is worth noting that many studies have reported rapid changes in the composition of the gut microbiota following dietary modifications. Dietary habits are major contributors to the diversity and changing composition of human gut microbiota. Therefore, dietary intervention may serve as an effective strategy for treating obesity by reducing energy intake and potentially modulating the gut microbiota to promote weight loss. However, the primary problem in treating patients with morbid obesity is maintaining long-term adherence to lifestyle and pharmacological interventions, which often results in insufficient weight loss despite initial success [28]. The involvement of gut microbiota in the development of obesity, as well as the precise mechanisms through which it influences obesity-related processes, remains under active investigation [19,29].

One of the key mechanisms linking gut microbiota to the development of obesity involves energy regulation and the microbiota’s capacity to ferment dietary polysaccharides. The anaerobic fermentation of dietary fibers and polysaccharides in the colon is facilitated by hydrolytic enzymes, which support carbohydrate breakdown and lead to the production of metabolites, including SCFAs. Bacterial species from the genera *Bacteroides*, *Roseburia*, *Ruminococcus*, *Bifidobacterium*, *Lactobacillus*, and *Eubacterium* represent important fiber-degrading bacteria [30]. SCFAs, primarily acetate, propionate, and butyrate in a molar ratio of 60:20:20, are metabolites and the relative amounts of each SCFA are influenced by the microbiota profile. Acetate is mainly produced by *Bifidobacteria* [30], while *Firmicutes* species such as *Faecalibacterium prausnitzii*, *Roseburia* spp., and *Eubacterium* species (e.g., *E. rectale* and *E. hallii*) are responsible for butyrate production [31]. Propionate is primarily produced by *Bacteroides*, *Prevotella*, and *Veillonella* [32]. SCFAs are involved in the interplay between gut microbiota, energy metabolism, diet, and the regulation of body weight [33]. Metagenomics-based studies indicate that the functional capabilities of the gut microbiota may be more significant than its composition [34,35].

SCFAs have been shown to indirectly influence metabolic pathways of the carbohydrate-response element binding protein (ChREBP) and the sterol regulatory element binding transcription factor 1 (SREBP1), which both induce lipogenesis and increase triglycerides (TG) stores. Additionally, SCFAs may lower the levels of fasting-induced adipocyte factor (FIAF), which suppresses lipoprotein lipase, resulting in TG deposition in host adipocytes [35].

Another mechanism linking gut microbiota to obesity is its capacity to reduce fatty acid oxidation in the liver by inhibiting adenosine monophosphate kinase (AMPK) [36]. AMPK acts as a master regulator of cellular energy metabolism, thermogenesis, and metabolic disorders. Activating hypothalamic AMPK stimulates appetite and promotes weight gain, while inhibiting it leads to weight loss [37,38]. The anti-obesity or anti-diabetic effects of medications used in clinical therapy, such as metformin, nicotinic acid, and liraglutide, act through AMPK. Therefore, AMPK is of interest from a therapeutic perspective [39].

Obesity and metabolic disorders are characterized by low-grade inflammation, that the gut microbiota may contribute by triggering systemic inflammation [40]. TLRs are immune cell membrane proteins that play a role in initiating intracellular signaling pathways, leading to the expression of cytokines and chemokines, which in turn influence the inflammatory response. Specifically, TLR4 binds LPS found in the cell membranes of Gram-negative bacteria within the gut microbiota and triggers sustained activation of pro-inflammatory mechanisms in the intestinal mucosal immune system. Intestinal dysbiosis observed in obesity contributes to low-grade inflammation and metabolic endotoxemia [41].

## 3. Postbiotics

In recent years, there has been growing attention toward alternative biotherapeutic agents composed of inanimated bacteria, microbial cell fragments, and their metabolic by-products. These substances are considered to offer certain benefits over traditional probiotics, particularly in terms of safety for fragile groups such as newborns, immunocompromised individuals, critically ill patients, and those with compromised intestinal barriers [42]. Such agents are collectively known as postbiotics, a term that has gained significant recognition in the scientific community.

The exact definition of postbiotics has long been debated and a number of different terms have been used, such as metabiotics, paraprobiotics or “ghost probiotics”, and fermented infant formula [42,43]. According to the latest definition proposed by the International Scientific Association for Probiotics and Prebiotics (ISAPP), a postbiotic is “a preparation of inanimate microorganisms and/or their components that confers a health benefit on the host” [43]. Postbiotics are substances, including metabolites and structural components of bacterial cell walls, that are generated by live bacteria or released following bacterial cell breakdown. Unlike probiotics, postbiotics do not involve the intake of live microorganisms and typically do not exert prolonged effects once the treatment is stopped [9]. Postbiotics exert various beneficial effects on the host, including antimicrobial, immunomodulatory, and antiobesogenic actions by influencing multiple biological pathways. These effects involve the modulation of lipid metabolism, and correction of gut dysbiosis, and exhibit anti-inflammatory, anticarcinogenic, antihypertensive, and antioxidant properties [44,45]. The well-known postbiotics include bacterial biologically active metabolic products, lysates from the degradation of Gram-positive and Gram-negative bacteria, antioxidant enzymes, exopolysaccharides (EPS) released by microbiota, cell wall components including teichoic acid, muropeptides derived from peptidoglycans (PGN), bacteriocins, vitamins, and short-chain fatty acids from degradation of polysaccharides by gut microbiota [46]. Individual postbiotics may exert beneficial effects by acting through one or more of several mechanisms, including (a) modulation of the host’s gut microbiota; (b) enhancement of epithelial barrier integrity; (c) regulation of both local and systemic immune responses; (d) influence on systemic metabolic processes; and (e) signaling via neural pathways [43].

Techniques for inactivating microorganisms to produce postbiotics can be broadly categorized into natural processes and laboratory-based methods, such as physical and chemical treatments. Among natural methods, fermentation stands out as a major source of postbiotic production. During fermentation, microbial cells metabolize prebiotic substrates resulting in the formation of various postbiotic compounds with distinct biological activities. This process enhances the nutritional and functional value of the food matrix [47]. Laboratory methods used for probiotic inactivation and postbiotic generation include thermal treatment, ionizing radiation, formalin exposure, ultraviolet (UV) irradiation, high-pressure processing, dehydration, supercritical carbon dioxide fluid technology, pH modification, sonication, omics-based approaches, and more recently, high-intensity ultrasound (HIUS) [48]. Inactivation of the viability of the parent microorganism must not lead to a loss of the original function of the postbiotic and must provide health benefits to the host [43].

### 3.1. Postbiotics with Anti-Obesity Effects in Preclinical Studies

Postbiotics may help prevent obesity through various mechanisms, such as enhancing energy expenditure, suppressing the formation and differentiation of adipocytes, decreasing food consumption, modulating lipid metabolism, and altering the composition of the gut microbiota [45,49,50]. Thus postbiotic supplementation could be a novel strategy for the treatment of obesity, which has gained significant attention from the scientific community. Table 1 summarizes various important postbiotics and their beneficial effects that have been shown to prevent and treat obesity in preclinical studies.

#### 3.1.1. Cell Wall Components

Cell wall components such as peptidoglycan, lipoteichoic acid (LTA), muramyl dipeptide (MDP), exopolysaccharides, and surface layer proteins (SLPs) represent important anti-obesity factors. Muramyl dipeptide modulates the release of glucagon-like peptide-1 (GLP-1), which affects appetite, increases energy expenditure, enhances fat oxidation, and improves glucose intolerance [51]. MDP supplementation reduced adipose inflammation and glucose intolerance in obese mice via nucleotide-binding oligomerization domain (NOD2) and interferon regulatory factor 4 (IRF4) independently of weight loss or changes in microbiome composition [52]. Surface layer proteins are attached to the peptidoglycan layer of the cell wall and significantly reduce the levels of inflammatory markers such as interleukin 6 (IL-6) and nuclear factor kappa B (NF-kB) in RAW 264.7 cells stimulated with lipopolysaccharides. Surface layer proteins reduced systemic inflammation, suppressed adipogenesis, and alleviated insulin resistance in mice fed a high-fat diet (HFD) [73]. LTA from *Lactobacillus* and other bacterial species reported immunomodulatory and anti-inflammatory properties [53,54]. LTA derived from *Bifidobacterium animalis* subsp. *lactis* BPL-1 has also been identified as a novel lipid modulator that exerts fat-reducing effects, potentially by acting through the insulin-like growth factor-1 (IGF-1) cell signaling pathway [55]. EPS produced by LAB (lactic acid bacteria) has shown antioxidant, anti-inflammatory, antiviral, antimicrobial, antitumor, and anti-biofilm activities. EPS from *L. rhamnosus* GG reduced hepatic and serum TG levels and reduced inflammation in HFD-fed mice [56]. EPS from *L. plantarum* L-14 extract suppressed the maturation of preadipocytes into adipocytes by activating the AMPK signaling pathway, leading to reduced expression of adiponectin and adipogenic markers, as well as a decrease in fat accumulation [57]. Cell wall components including EPS, and SLP as postbiotics from kefir LAB in combination with a prebiotic (grape seed flour) demonstrated a synergistic antiobesitogenic effect by improving chronic inflammation, adipogenesis, glucose intolerance along with alteration of the gut microbiota in animals fed with HF diet [74].

#### 3.1.2. Biotransformation (Bioconversion) Products

Bioconversion refers to the transformation of chemical compounds through structural modification, with microbes serving as a key tool for this process in the food industry. LAB have traditionally been employed as starter cultures in dairy products and plant-based fermented foods [75]. Lee et al. [58] reported that postbiotics derived from whey medium biotransformed by *Pediococcus pentosaceus* KI31 and *Lactobacillus sakei* KI36 prevented the differentiation of 3T3-L1 preadipocytes, as evidenced by reduced intracellular lipid accumulation and down-regulation of the transcription factor peroxisome proliferator-activated receptor γ (PPAR- γ) and the genes for adipocyte fatty acid-binding protein (A-FABP) and lipoprotein lipase.

The transformation of whey and polyphenol-rich citrus pomace extract with *Lactobacillus kefiri* DH5 resulted in bioactive postbiotics that improved the ratio of adipose tissue weight to body weight, increased gene expression associated with energy expenditure in adipose tissue, TG level, adipocyte size, and increased the number of butyrate-producing bacteria *Olsenella profusa* and *Anaerovorax odorimutans* in C57BL/6J mice on high-fat diets [59].

#### 3.1.3. Cell-Free Lysates

In the context of postbiotics, cell-free lysates are non-viable microbial preparations that provide health benefits and mimic the effects of probiotics after a cell inactivation procedure. They offer a more controlled way to deliver microbial-derived bioactivity [9,76]. In experimental studies, cell lysates obtained by heat inactivation are commonly used, with beneficial effects that include immunomodulation, protection against enteropathogens, and maintenance of intestinal barrier integrity [77]. However, several animal studies reported also beneficial effects of cell-free lysates in obesity management. Postbiotics from L. paracasei were applied to rats with HFD-affected weight and lipid values. Atorvastatin and postbiotics prevented body weight gain demonstrated a lipolytic effect, and reduced levels of TG and total cholesterol (TC) [60]. Supplementation of heat-treated strain *L. plantarum* L-137 showed a reduction in lipopolysaccharide-binding protein (LBP) levels, an important marker of intestinal permeability, reduced adipose tissue inflammation, partially increased intestinal permeability, and reduced endotoxin translocation [61].

#### 3.1.4. Bacterial Extracellular Vesicles

Bacterial extracellular vesicles (BEVs) are products derived from microorganisms and contain proteins, polysaccharides, lipids, enzymes, and toxins. These components can interact with host cells, influence immune responses, modulate intestinal permeability, and even impact systemic metabolism [78]. Dysfunction of the intestinal barrier is associated with obesity. The bacteria *Akkermansia muciniphila* is important for maintaining homeostasis of intestinal barriers and intestinal health [79]. EVs obtained from *A. muciniphila* showed efficacy against obesity, pasteurized *A. muciniphila* reduced adipose tissue accumulation in HF diet, liver inflammation, increased expression of genes for maintaining homeostasis and lipid metabolism, and improved gut dysbiosis [62]. Chronic low-grade inflammation is a hallmark of obesity. Several preclinical studies showed that BEVs from probiotic strains such as *Propionobacterium freudenreichii* CIRM-BIA 129, *Lacticaseibacillus rhamnosus* JB-1, or *Bacteroides thetaiotaomicron* are able to suppress the pro-inflammatory pathway of NF-kB and lead to overexpression of cytokines TNF-α and IL-6, while also stimulating anti-inflammatory pathways (e.g., IL-10) [63,64,65]. Anti-inflammatory activities of postbiotics may contribute to improved insulin sensitivity and glycemic control [80,81].

#### 3.1.5. Bacteriocins

Bacteriocins are generally antimicrobial proteinaceous substances, typically peptides, produced by both Gram-positive and Gram-negative bacteria. They are known for their adequate heat stability and safety, and are widely used in the food sector as postbiotics. [82]. Despite the main function of bacteriocins as antimicrobial compounds several preclinical studies reported their beneficial effects in alleviating obesity and related parameters. Plantaricin EF—a bacteriocin from *L. plantarum* in obese mice was evaluated as an anti-obesity agent, causing a decrease in body weight and food intake while preserving the integrity of the epithelial barrier without changing microbiota composition [66]. Mahdavi et al. [67] showed that gassericin A produced by *L. gasseri* LA39 reduced serum cholesterol, LDL, and liver enzymes and improved the redox status of mice fed both high-fat and high-sugar diets. In addition, bacteriocin decreased the expression of specific obesity-associated genes Zfp423 and Fabp4 in abdominal adipose tissue. Despite these beneficial changes, there was no reduction in weight gain and abdominal fat after 10 weeks of postbiotic intervention.

#### 3.1.6. SCFAs

SCFAs are the best-studied postbiotics derived from the gut microbiota. These metabolites are produced by anaerobic bacterial fermentation of prebiotics, polysaccharides, and fiber in the colon, and have been proposed as potential disease-modifying factors and facilitators of obesity prevention [83].

These acids play a preventive role in fat accumulation in adipose tissue, regulating energy intake and expenditure, as well as the production of satiety hormones, which help reduce body weight and improve metabolic parameters. The content and profile of SCFAs are influenced by the composition of the gut microbiota, with Firmicutes often associated with butyrate production, while Bacteroidetes mainly produce acetate and propionate [33]. SCFAs can affect the gut and body through both direct and indirect mechanisms. Direct actions involve SCFAs impacting enterocytes to maintain the integrity of the intestinal barrier, promote the formation of tight junction proteins (TJs), stimulate the production of mucin and antimicrobial peptides, suppress the formation of lipopolysaccharides, and lower the pH of the intestinal contents. Indirectly, SCFAs regulate the inflammatory response by modulating the immune system, as well as energy intake, glucose, and lipid metabolism [33,84,85].

Indirect actions are carried out through three mechanisms:

(a) SCFAs exert an anti-inflammatory effect by inhibiting histone deacetylase (HDAC) activity, leading to increased histone acetylation, which alters gene expression by promoting the availability of transcription factors in promoter regions [86];
(b) The initiation of signal transduction in various organs and the stimulation of the intestinal hormone release, such as glucagon-like peptide-1 (GLP-1) and peptide YY (PYY), occur following SCFAs binding to G protein-coupled receptors (GPCRs). Acetate, the most abundant SCFA, positively impacts host energy metabolism by influencing gut hormones like GLP-1 and PYY. Once in the bloodstream, these hormones affect appetite, reduce lipolysis, lower pro-inflammatory cytokines, and increase energy expenditure and fat oxidation [85];
(c) Butyrate acts as a ligand, or signaling molecule, for the aryl hydrocarbon receptor (AhR) and PPARγ, both of which are transcription factors that regulate gene expression. By activating these receptors, butyrate modulates gene expression and influences various aspects of gut health, metabolism, and immune responses [87].

SCFAs act as signaling molecules, activating specific receptors on intestinal and other tissue cells, triggering intracellular signaling cascades. These receptors include free fatty acid receptor 3 (FFAR3 or GPR41), FFAR2 (GPR43), G-coupled receptor protein 109a (GPR109a or HCAR2), olfactory receptor 78 (Olfr78) in mice or olfactory receptor 51E2 receptor in humans (OR51E2), GPR42, and OR51E1. FFAR3, FFAR2, and GPR109a receptors are found in various tissues and cells, including the gastrointestinal tract, skeletal muscle, heart, immune cells, and nervous system. FFAR2 (GPR43) receptor is highly expressed in immune cells and preferentially binds to acetate and propionate. FFAR3 is predominantly expressed in adipocytes, while GPR109a (HCAR2), a receptor for butyrate and niacin, is primarily expressed in intestinal epithelial cells [84]. Olfr78, a receptor primarily activated by acetate and propionate, plays a role in blood pressure regulation by modulating renin release in vascular smooth muscle cells and renal afferent arterioles. Activation of Olfr78 leads to an increase in cyclic adenosine monophosphate (cAMP) levels, which subsequently triggers the release of renin [33]. Butyrate and propionate regulate body weight by influencing food intake, likely through their ability to stimulate the release of anorexigenic (appetite-suppressing) gut hormones, which signal to the brain that you’re full. They also protect against diet-induced obesity [70].

The effects of SCFAs, individually or in combination, contribute to improving the integrity of the intestinal barrier.

SCFAs are essential substrates for maintaining colonic epithelial functions. Butyrate is a key energy source for normal colonocytes, supporting their proliferation. However, it also exerts a distinct effect on cancerous colonocytes, inducing cell cycle arrest, differentiation, and apoptosis. Butyrate improves intestinal barrier integrity by increasing the expression of tight junction proteins such as claudin-1, claudin-7, zonulin-1, and zonulin-2 [88]. Proper integrity of the intestinal barrier is essential to prevent pathogens from entering the bloodstream and various tissues, where they can promote metabolic disorders by influencing the immune system and regulating the host’s metabolic and inflammatory responses. Additionally, butyrate can stimulate goblet cells to increase the production of mucin glycoproteins, which play a vital role in maintaining the integrity of the intestinal barrier [85]. SCFAs can promote the secretion of antimicrobial peptides by intestinal epithelial cells and stimulate the production of regenerating islet-derived protein III gamma and defensins by activating mTOR (serine/threonine protein of signaling cascade) and STAT3 (signaling transducer and activator of transcription 3). Both signaling factors are involved in various cellular processes and contribute to the regulation of epithelial barrier functions [89].

- the regulation of lipid metabolism

SCFAs regulate lipid catabolism and adipogenesis. Acetate and propionate, in particular, can reduce endogenous lipolysis. They have been shown to inhibit lipolysis, particularly in adipocytes. Propionate can influence extracellular lipolysis by upregulating the expression of lipoprotein lipase. Increased activity of lipoprotein lipase leads to lower levels of circulating lipids in the blood plasma and may potentially promote weight loss. Acetate, propionate, and butyrate can enhance the absorption of cholesterol from the blood by the liver, thereby reducing plasma cholesterol levels. Furthermore, propionate itself is a potent inhibitor of cholesterol synthesis [33,69].

- improving glucose homeostasis and insulin resistance

SCFAs may have antidiabetic effects in the host by improving glucose homeostasis, mediated by activation of FFAR2 and FFAR3 [90]. Although the mechanisms are not entirely clear, these effects may occur either directly via AMPK activation or indirectly through the hormones GLP-1 and PYY [70]. PYY is involved in glucose metabolism in adipose and muscle tissues, while GLP-1 contributes to blood glucose regulation by promoting insulin secretion and inhibiting glucagon release from the pancreas [91].

- the regulation of energy intake and expenditure

SCFAs have positive effects on body weight regulation and appetite, contributing to the balance between energy intake and expenditure. A potential mechanism involves the stimulation of the gut hormones PYY and GLP-1, which are secreted by enteroendocrine L cells in the intestine. The release of these hormones may be initiated by the binding of SCFAs to the FFAR2 and FFAR3 receptors on L cells. Both GLP-1 and PYY regulate appetite by activating proopiomelanocortin neurons in the hypothalamic arcuate nucleus, suppressing neuropeptide Y-producing neurons, and slowing gastric transit [92]. Additionally, SCFAs stimulate the secretion of leptin, often referred to as the “satiety hormone”, which leads to a decrease in appetite [93].

- the regulation of the immune system and anti-inflammatory reactions

SCFAs modulate inflammation by regulating cytokine production. They contribute to the anti-inflammatory effect by suppressing pro-inflammatory factors and increasing the production of anti-inflammatory cytokines [94]. In intestinal epithelial cells, SCFAs induce the release of interleukins 18 (IL-18), which helps maintain intestinal homeostasis. Butyrate and propionate decrease the secretion of pro-inflammatory chemokines and downregulate the expression of cytokines such as interleukin-6 (IL-6) and interleukin-12p40 (IL-12p40), particularly in response to LPS stimulation [95,96]. By regulating cytokine production and lowering luminal pH, SCFAs inhibit the growth of pathogenic bacteria. Butyrate supports host defense by promoting the production of the active antimicrobial peptide LL-37, derived from cathelicidin, and by enhancing the population of regulatory T cells in the intestine [97].

- the regulation of blood pressure

SCFAs may be effective in regulating hypertension by improving glucose homeostasis [98].

#### 3.1.7. Other Metabolites

Urolithins (Uro) are bioactive substances that are formed following intense intestinal microbial activity on ellagitannins and ellagic acid. There are several types of urolithins (Uro-C, Uro-A, Uro-D, Uro-B), of which Uro-A and Uro-B are the most biologically effective [99]. Administering these compounds to rats fed an HFD produced anti-obesogenic effects, marked by an increase in *Parabacteroides* and a reduction in *Desulfovibrionaceae* and *Coriobacteriaceae*, which were associated with weight loss and beneficial alterations in lipid metabolism. The underlying molecular mechanism involves promoting the browning of white adipose tissue and enhancing thermogenesis in brown adipose tissue, resulting in higher energy expenditure [72].

## 4. Effectiveness of Postbiotics in Clinical Studies

The use of postbiotics to mitigate risk factors for obesity and metabolic disorders has so far only been confirmed in a limited number of human studies, which are summarized in Table 2.

Acute administration of inulin-propionate ester in overweight individuals (n = 60) significantly increased the secretion of postprandial PYY and GLP-1 and reduced calorie intake. Long-term intake of inulin-propionate ester led to reduced calorie intake, reduced weight gain and distribution in intra-abdominal adipose tissue, and also decreased intrahepatocellular lipid content [100]. Van der Beek et al. examined the impact of administering acetic acid to the distal colon in overweight and obese individuals [101]. In the treatment group colon acetate increased fasting fat oxidation, PYY concentration, insulin concentration, and decreased tumor necrosis factor-alpha compared to the placebo group. Tested colonic infusion of SCFA mixture (propionate, acetate, butyrate) in obese normoglycaemic men significantly increased fasting fat oxidation, PYY, resting energy expenditure, and decreased lipolysis [102]. Dietary fiber intake can influence the composition of gut microbiota, particularly those that produce SCFAs. After a three-month high-fiber diet, patients with type 2 diabetes mellitus showed an increased abundance of SCFA-producing bacteria, such as *F. prausnitzii* and *A. muciniphila*, along with reductions in blood glucose, total cholesterol, LDL cholesterol, free fatty acids, and HbA1c levels. These findings suggest that long-term consumption of a high-fiber diet may improve glycemic control, balance dyslipidemia, and decrease inflammation by promoting the growth of SCFA-producing gut microbes [111]. The impact of sodium butyrate and inulin supplementation, both individually and together, on glycemic control, lipid profile, and GLP-1 levels was investigated in adults with type 2 diabetes mellitus. Dietary supplementation significantly reduced diastolic blood pressure in comparison with the placebo. However, only the combination of sodium butyrate and inulin significantly decreased fasting blood sugar and waist-to-hip ratio. The concentration of GLP-1 was significantly increased in groups with sodium butyrate and groups with their combination in comparison to placebo. The findings indicate that inulin supplementation could be advantageous for diabetic patients, with its effects potentially enhanced by butyrate supplementation [103].

The Butyrate Against Pediatric Obesity (BAPO) study was conducted to assess the potential benefits of butyrate supplementation as a treatment for childhood obesity. Obese children treated with butyrate experienced a greater decrease in BMI, along with beneficial changes in fasting glycemia, lipid parameters, ghrelin level, IL-6, and HOMA-IR. Dietary and lifestyle habits have changed the gut microbiome [104].

In overweight/obese men, the bioaccessibility of two different SCFA-enriched triglycerides (Akovita SCT and tributyrin/caproin) was compared using an in vitro model of the stomach and small intestine (TIM-1). The esterification of SCFA-enriched triglycerides with long-chain fatty acids slowed the release of SCFAs from the glycerol backbone. Akovita SCT elevated postprandial levels of circulating butyrate and hexanoate without affecting metabolic parameters [105]. The study of Amiri et al. [106] revealed that sodium butyrate supplementation alone had no significant effects on anthropometric and biochemical parameters in obese individuals. These parameters change when sodium butyrate is combined with calorie restriction.

The anti-obesity effects of postbiotic *Pediococcus pentosaceus* LP28 from longan fruit were tested on overweight subjects as a placebo (LP28 live) or heat-killed LP28 for 12 weeks. Significant reductions in body fat percentage, waist circumference, BMI, and body fat mass were observed in the group supplemented with the LP28 postbiotic [107]. The impact of heat-inactivated and lyophilized bacterial powder derived from *Lactobacillus amylovorus* CP1563 on lipid metabolism was assessed in subjects with a BMI of 25–30 kg/m^2^ over 12 weeks [108]. Supplementation with *Lactobacillus amylovorus* CP1563 significantly reduced total fat mass, body fat percentage, and visceral adipose tissue, as well as the concentrations of circulating lipids such as TG, TC, and LDL-C, and also improved diastolic blood pressure levels. In another study, postbiotic *L. amylovorus* CP1563 affected the gut microbiota through significantly higher counts of *Roseburia* and *Lachnospiraceae* compared to placebo [109].

In individuals with abdominal obesity, consumption of seafood sticks enriched with postbiotics (heat-inactivated *Bifidobacterium animalis* subsp. *lactis* CECT8145) and bioactive components (including the prebiotic inulin and omega-3 fatty acids) demonstrated potential protective effects against the development of type 2 diabetes. These effects were reflected in decreased insulin levels, improved HOMA-IR (Homeostatic Model Assessment of Insulin Resistance), lower pulse pressure, and reduced postprandial concentrations of atherogenic TG. Biochemical improvements were partially linked to alterations in the composition of the gut microbiota. The results showed the effectiveness of enriched seafood sticks in the management of cardiometabolic risk factors in obese individuals [110].

According to the published results of human clinical studies, postbiotics such as SCFAs and heat-inactivated probiotics show promising potential in improving body composition and metabolic health in obesity. However, the application of postbiotics in the treatment of obesity is still at an early stage. This is supported by only four clinical trials registered in the online database of clinical research studies, ClinicalTrials.gov. Ongoing trials are evaluating postbiotic supplementation in promoting weight loss and metabolic health in obese patients with type 2 diabetes (DELI_Diab Study—NCT05770076) and in obese children and adolescents (PostOb—NCT06309121, KOBI—NCT05428137, NCT06911073) [112].

## 5. Conclusions

Obesity remains a rapidly escalating global health issue. As a complex, multifactorial disease, recent research highlights the gut microbiota as a critical player in its underlying mechanisms. The gut microbial community can influence obesity through several pathways, such as regulating energy balance, triggering inflammation via LPS, altering bile acid metabolism, and affecting fat storage. While targeting the gut microbiota shows promise for obesity prevention, the specific bacterial populations involved in its onset and progression are still not fully understood. Modulation of the gut microbiota is increasingly considered a promising strategy in the management of obesity, though it is still an evolving area of research. Unlike live probiotics, postbiotics offer enhanced safety and stability while still exerting beneficial effects on host metabolism, inflammation, and gut microbiota composition. Preclinical studies suggest that postbiotics can modulate adipogenesis, improve insulin sensitivity, and reduce systemic inflammation—all key factors in the pathophysiology of obesity. Although the use of postbiotics in obesity management is still in its early stages, a limited number of clinical trials have indicated their potential benefits. However, more extensive, long-term, and well-designed clinical trials are needed to confirm the clinical efficacy of postbiotics, determine optimal formulations, and integrate them effectively into dietary or therapeutic protocols. Therefore, this remains a relatively novel topic in clinical research.

## Figures and Tables

**Table 1:** Postbiotics and their anti-obesogenic effects in preclinical studies.

Postbiotic Molecules	Origin	Preclinical Model	Anti-Obesogenic Effects	Ref.
Cell wall components				
Muramyl dipeptide		murine GLUTag and human NCI-H716 cells C57BL/6J mice on HFD	↑ glucose tolerance by stimulating GLP-1 secretion via activation of the NOD2 pathway ↓ adipose inflammation and glucose intolerance via NOD2 and IRF4	[51] [52]
Lipoteichoic acid	*Laciplantibacillus* *plantarum* CRL1506 *Lacticaseibacillus* *paracasei* 6–1 *Bifidobacterium animalis* subsp. *lactis* BPL1	porcine epithelial cells RAW 264.7 macrophages *Caenorhabditis elegans*	anti-inflammatory properties—↓ expression of IL-6 and MCP-1 anti-inflammatory effects through ↓ TLR4-MyD88-MAPK and NF-κB signaling pathways fat-lowering effect through IGF-1 signaling pathway in hyperglycemic conditions	[53] [54] [55]
Exopolysaccharides	*Lacticaseibacillus* *rhamnosus* GG *Lactiplantibacillus* *plantarum* L-14	3T3-L1 preadipocytes C57BL/6J mice on HFD C57BL/6J mice on HFD	↓ lipid accumulation in adipocytes through TLR2 signalisation ↓ hepatic and serum TG ↓ inflammation—↓ IL-6, MCP-1 ↓ gene expression of markers of M1-like macrophages ↓ early stage of adipogenic differentiation by upregulating AMPK signaling pathway, ↓ expression of adipogenesis markers PPARγ, C/EBPα, FABP4 ↓ TG/HDL ratio and steatohepatitis ↓ pro-inflammatory molecules leptin, IL-6, TNF-α and resistin ↑ expression of anti-inflammation markers adiponectin and Arg1	[56] [57]
Biotransformation (bioconversion) products				
Whey medium	biotransformed by *Pediococcus pentosaceus* KI31 and *Lactobacillus sakei* KI36	3T3-L1 preadipocytes	↓ differentiation of preadipocytes and intracellular lipid accumulation ↓ PPAR- γ and the genes for A-FABP and lipoprotein lipase	[58]
Whey and polyphenol- rich citrus pomace extract	biotransformed by *Lactobacillus kefiri* DH5	C57BL/6J mice on HFD	improved the adipose tissue weight/body weight ratio ↑ expression of the genes related to energy expenditure UCP-1 and PGC-1α in adipose tissue ↑ number of butyrate-producing bacteria *Olsenella profusa, Anaerovorax odorimutans*	[59]
Cell-free lysates				
	*L* *acticaseibacillus paracasei*	Wistar rats on HFD	↓ total serum lipids, TG and total cholesterol ↑ AST and ALT—improvement of liver function and antioxidant enzymes—glutathione peroxidase, glutathione-s-transferase, glutathione reductase, superoxide dismutase	[60]
	*Lactiplantibacillus* *plantarum* L-137	C57BL/6 J mice on HFD	↓ weight gain; improvement of metabolism—↓ glucose, cholesterol, alanine aminotransferase, and aspartate transaminase levels; ↓ LBP—improvement of intestinal permeability; ↓ adipose tissue inflammation—↓ expression of F4/80, CD11c, and IL-1β	[61]
Bacterial extracellular vesicles				
	*Akkermansia muciniphila*	C57BL/6 mice on HFD	↓ food intake and body and adipose weight gain; ↑ expression of genes PPAR-α, and PPAR-γ involved in fatty acid oxidation and energy metabolism in adipose tissue ↓ inflammation associated with ↓TNF-α, IL-6 and TLR-4 expression in adipose tissue; ↓ intestinal permeability—↑ expression of ZO-1, OCLDN and CLDN-1	[62]
	*Propionobacterium freudenreichii* CIRM-BIA 129,	HT-29 cell line	are able to suppress the pro-inflammatory pathway of NF-kB and lead to overexpression of cytokines TNF-α and IL-6, while also stimulating anti-inflammatory pathways (e.g., IL-10)	[63]
	*Lacticaseibacillus* *rhamnosus* JB-1	murine BMDCs	activation of TLR2 and ↑ expression of immunoregulatory IL-10	[64]
	*Bacteroides thetaiotaomicron*	SPF C57BL/6 mice with DSS induced colitis murine BMDCs	upregulation of IL-10 production in colonic tissue ↑ ratio of IL-10/TNFα	[65]
Bacteriocins				
Plantaricin EF	*Lactiplantibacillus plantarum*	C57BL/6J mice on HFD	↓ body weight and food intake; improved oral glucose tolerance ↓epithelial barrier permeability—↑ expression of ZO-1	[66]
Gassericin A	*Lactobacillus gasseri* LA39	C57Bl/6J mice on HFD	↓ serum cholesterol, LDL, liver enzymes and improved the redox status ↓ expression of specific obesity-associated genes Zfp423 and FABP4 in abdominal adipose tissue.	[67]
Short chain fatty acids				
Acetate, propionate butyrate	dietary	C57Bl/6J mice on HFD	↓ PPARγ expression and ↑ oxidative metabolism in liver and adipose tissue via AMPK; ↓ body weight and hepatic steatosis; improving insulin sensitivity	[68]
Acetate, propionate butyrate	dietary	C57BL/6 J mice on HFD	↓ body weight reduction ↑ TG hydrolysis and FFA oxidation in the adipose tissue, ↑ expressions of GPR43 and GPR41 in the adipose tissue modulation of gut microbiota composition— ↓ Firmicutes and ↑ Bacteroidetes.	[69]
Propionate, butyrate	dietary	C57BL/6N mice on HFD	↓ food intake, weight gain and insulin resistance ↑ of anorexigenic peptides GLP-1, PYY, and amylin	[70]
Acetate, propionate, butyrate		bovine adipocytes	↑ leptin expression	[71]
Other metabolites				
Urolithins	dietary	Wistar rats on HFD	↓ bodyweight, serum levels of cholesterol, TG and LDL-C ↑ HDL-C. ↑ increased the relative abundance of *Parabacteroides* and ↓ *Coriobacteriaceae* and *Desulfovibrionacea*	[72]

Abbreviations: GLP-1: glucagon like peptide-1, HFD: high fat diet, NOD2: nucleotide binding oligomerization domain containing 2, IRF4: interferon regulatory factor 4, IL-6: interleukin 6, MCP- 1: monocyte chemoattractant protein 1, TLR: Toll like receptor, MyD88: MYD88 innate immune signal transduction adaptor, MAPK: mitogen activated kinase-like protein, NF-κB: nuclear factor kappa B, IGF-1: insulin like growth factor 1, TG: triglycerides, AMPK: 5′ adenosine monophosphate-activated protein kinase, PPAR: peroxisome proliferator activated receptor, C/EBPα: CCAAT enhancer binding protein alpha, FABP4: fatty acid binding protein 4, HDL: high-density lipoprotein cholesterol, TNF-α: tumor necrosis factor α, Arg1: arginase 1, A-FABP: adipocyte-type fatty acid-binding protein, UCP-1: uncoupling protein-1, PGC-1α: peroxisome proliferator-activated receptor gamma coactivator-1α, AST: aspartate aminotransferase, ALT: alanine transaminase, LBP: lipopolysaccharide binding protein, IL-1: interleukin 1, BMDC: bone marrow–derived dendritic cell, ZO-1: zonulin 1, OCLD: ocludine, CLDN-1: claudine 1, Zfp423: zinc finger protein 423, FFA: free fatty acid receptor, GPR 43: G-protein coupled receptor 43, PYY: peptide YY, ↓ = Decrease, ↑ = Increase.

**Table 2:** Antiobesogenic effects of postbiotics in clinical trials.

Design/Target Population	Type of Postbiotics/Dose	Duration of Intervention	Control/Dose	Results	Ref.
**Short Chain Fatty Acid** **s (SCFAs)**					
Randomized, double-blind, placebo-controlled, parallel design/overweight adults BMI: 25–40 kg/m^2^ (n = 60)	Inulin-propionate ester/10 g/day	24 weeks	inulin/ 10 g/day	↓calorie intake ↓weight gain ↓intra-abdominal adipose tissue distribution ↓intrahepatocellular lipid content ↑PYY and GLP-1 secretion ↓ LDL-C and AST	[100]
Randomized, double-blind, crossover trial/overweight/obese men BMI: 25–35 kg/m^2^ (n = 6)	Sodium acetate/(100 or 180 mmol/L dissolved in 120 mL 0.9% NaCl Two experimental periods: one with distal and one with proximal colonic sodium acetate infusions.	3 days	Placebo: 120 mL 0.9% NaCl	Distal colonic acetate: ↑Fasting fat oxidation ↑PYY ↑postprandial glucose ↑insulin concentration ↓TNF-α Proximal colonic acetate: no effects on substrate metabolism, circulating hormones, or inflammatory markers	[101]
Randomized, double-blind, crossover study/ normoglycaemic overweight/obese men BMI: 25–35 kg/m^2^ (n = 13)	SCFA mixtures high in either acetate (HA), propionate (HP), butyrate (HB)/ HA solution: 24 mmol Na acetate (60%), 8 mmol Na propionate (20%), 8 mmol Na butyrate (20%) HP solution: 18 mmol Na acetate (45%), 14 mmol Na propionate (35%), 8 mmol Na butyrate (20%) HB solution: 18 mmol Na acetate (45%), 8 mmol Na propionate (20%), 14 mmol Na butyrate (35%) all in 200 mL water	4 days	Placebo: 40 mmol sodium chloride in 200 mL water	All three SCFA mixtures: ↑fasting fat oxidation ↑PYY (fasting and postprandial plasma) ↓lipolysis After HA and HP compared with placebo: ↑resting energy expenditure	[102]
Randomized, double-blind, placebo-controlled clinical trial/ adults with type 2 diabetes mellitus BMI: 27–35 kg/m^2^ (n = 60)	Sodium butyrate (capsules) and inulin (powder) supplementation alone or in combination/ group A—sodium butyrate (600 mg/d) group B—inulin (10 g/d) group C—sodium butyrate (600 mg/d) and inulin (10 g/d)	45 days	group D—Placebo: 600 mg starch capsules as well as 10g of starch powder	Treatment with sodium butyrate + inulin (group C): ↓fasting blood sugar and WHR After intervention in groups B and C: ↓WC Treatment in group A and C: ↑GLP-1 in comparison with group D	[103]
Randomized, quadruple-blind, placebo-controlled trial/children (age 5–17 years, BMI > 95th percentile (n = 54)	Sodium butyrate (capsules)/ 20 mg/kg body weight per day	6 months	cornstarch capsules	↓BMI and WC ↓HOMA-IR and fasting insulin level ↓microRNA221 relative expression ↓ghrelin and IL-6 level	[104]
Double-blind placebo-controlled randomized crossover study/ overweight/obese men BMI: 25–35 kg/m^2^ (n = 12)	A liquid high-fat mixed meal containing either a low (650 mg), medium (1325 mg), or high (2000 mg) dose of butyrate and hexanoate-enriched triglycerides—Akovita SCT (order in which the doses were received was randomized)	4 days	A liquid high-fat mixed meal containing the placebo: sunflower oil	The medium and high doses of Akovita SCT: ↑ postprandial circulating butyrate and hexanoate Akovita SCT supplementation did not affect subjective appetite, GLP-1 release, metabolic parameters, or inflammatory markers compared to placebo.	[105]
Triple-blind placebo-controlled randomized clinical trial/ obese adults BMI: 30–40 kg/m^2^ (n = 50)	Sodium butyrate (capsules) + hypo-caloric diet/ 600 mg/d NaB + diet included carbohydrates 55–60%, fat 25–30%, and protein 10–15% of total energy expenditure	60 days	Placebo: 600 mg carboxymethyl cellulose	↓BMI, weight, WHR and WC ↑PGC-1α and UCP-1 genes expression ↓ FBS, LDL–C and HDL-C	[106]
**Cell-free lysates**					
Randomized, double-blind, placebo-controlled study/ healthy adults BMI: 25–30 kg/m^2^ (n = 62)	living *Pediococcus pentosaceus* LP28 (powder) with dextrin, heat-killed *Pediococcus pentosaceus* LP28 (powder) with dextrin	12 weeks	Placebo: dextrin only	↓BMI, WC, body fat percentage and body fat mass not change fasting plasma glucose, HbA1c, fasting insulin, HOMA-IR, serum lipid levels	[107]
Randomized, double-blind, placebo-controlled clinical trial/ healthy overweight and pre-obese adults BMI: 25–30 kg/m^2^ (n = 200)	fragmented *Lactobacillus amylovorus* CP1563/ 200 mg in a 500 mL bottle per subject per day	2 weeks observation before treatment, 12 weeks treatment, and 4 weeks observation after treatment	Placebo: 500 mL bottle of the beverage per volunteer per day	↓body fat percentage, visceral fat and whole body fat ↓ total cholesterol, triglycerides and LDL–C ↓ diastolic blood pressure ↓ plasma glucose, insulin and HOMA-IR ↓ Uric acid	[108]
Randomized, double-blind, placebo-controlled, parallel-group study healthy adults BMI: 25.0–29.9 kg/m^2^ (n = 169)	fragmented *Lactobacillus amylovorus* CP1563 and 10- hydroxyoctadecanoic acid (10-HOA)/ 500 mL bottle of the beverage with the fragmented CP1563 containing 10-HOA per subject per day	12 weeks	Placebo: 500 mL bottle of the beverage without the fragmented CP1563 per subject per day	↓ abdominal visceral fat area, subcutaneous fat area, and total fat area ↓BMI and body weight ↑ genera *Roseburia* and *Lachnospiraceae* ↓ genus *Collinsella*	[109]
Randomized, double-blind, placebo-controlled, parallel-group study abdominally obese adults BMI: 25.0–29.9 kg/m^2^ (n = 120)	Heat-treated *Bifidobacterium animalis* subsp.lactis CECT 8145 SIAP2, 50 g/day conventional seafood sticks + heat-treated *Bifidobacterium animalis* subsp. lactis CECT 8145 + 370 mg/day EPA and DHA + 1.7 g/day inulin	12 weeks	Placebo: 50 g/day conventional seafood sticks	↓ insulin and HOMA-IR ↓ pulse pressure in women SIAP2 consumption: negative association between glycemic parameter reduction and *Alistipes finegoldii* and Ruminococcaceae. In the acute single dose-study 4-h, SIAP2 consumption: ↓ postprandial circulating triglyceride	[110]

Abbreviations: BMI: body mass index, PYY: peptide YY, GLP-1: glucagon-like peptide-1, LDL-C: low-density lipoprotein cholesterol, AST: aspartate aminotransferase, TNF-α: tumor necrosis factor -α, SCFA: Short Chain Fatty Acid, WHR: waist-to-hip ratio, WC: waist circumference, HOMA-IR: homeostasis model assessment of insulin resistance, PGC-1α: peroxisome proliferator-activated receptor gamma coactivator-1α, UCP-1: uncoupling protein-1, FBS: fasting blood sugar, HDL-C: high-density lipoprotein, HbA1c: Hemoglobin A1C, NaB: sodium butyrate, ↓ = Decrease, ↑ = Increase.

## Data Availability

The data contained in the article are derived from public domain resources.
